# NMR spectra and electrochemical behavior of catechol-bearing block copolymer micelles

**DOI:** 10.1016/j.dib.2015.04.004

**Published:** 2015-04-22

**Authors:** Urara Hasegawa, Masaki Moriyama, Hiroshi Uyama, André J. van der Vlies

**Affiliations:** a Frontier Research Base for Young Researchers, Graduate School of Engineering, Osaka University, 2-1, Yamadaoka, Suita 565-0871, Osaka, Japan; b Department of Applied Chemistry, Graduate School of Engineering, Osaka University, 2-1, Yamadaoka, Suita 565-0871, Osaka, Japan; c Frontier Research Center, Graduate School of Engineering, Osaka University, 2-1, Yamadaoka, Suita 565-0871, Osaka, Japan

**Keywords:** Polymer, Catechol, Redox

## Abstract

Here, we provide the NMR spectra and AFM data for antioxidant micelles prepared from amphiphilic PAM-PDA block copolymers composed of a poly(*N*-acryloyl morpholine) and a redox-active catechol-bearing block with different catechol content. We also provide details of the electrochemical analysis that showed micelles higher catechol content had a similar redox potential with the small catechol compound dopamine, but slowed down the redox reaction (Hasegawa et al., Polymer (in press)).

Specifications table

**Table:** 

Subject area	*Chemistry*
More specific subject area	*Polymeric micelles with antioxidant activity*
Type of data	*Spectra, table, image (microscopy), graph*
How data was acquired	*NMR, cyclic voltammetry, Atomic force microscopy*
Data format	*Raw*
Experimental factors	*The PAM-PDA block copolymers in DMF were dispersed in acetate buffer (pH5.0) followed by dialysis against water to prepare the micelles.*
Experimental features	*Characterization of polymer, polymeric micelles and their electrochemical property*
Data source location	*Osaka University*
Data accessibility	*The data is available with this article and is related to*[1]

Value of the data

• Antioxidant micelles with different structural and oxidation stability were prepared from amphiphilic block copolymers composed of a PAM block and a catechol-bearing block.
• Electrochemical behavior of the micelles having different structural stability was investigated at different pH.
• Incorporation of catechol moieties within the micelle core did not affect their redox potential compared to the small catechol compound dopamine, but slowed down the redox reaction.

## 1 Data

### 1.1 NMR spectra of the block copolymers

**Fig. 1:**
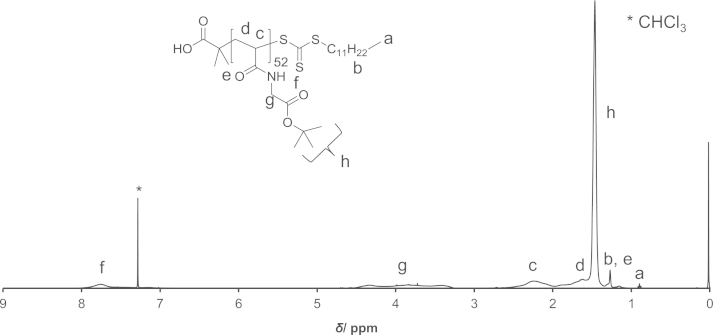
^1^H NMR spectrum of PAG polymer (**3**).

**Fig. 2:**
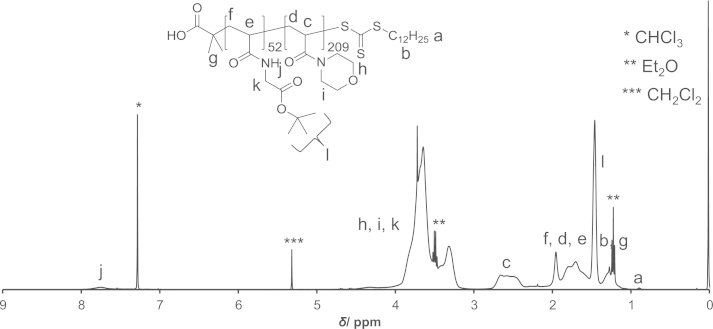
^1^H NMR spectrum of polymer (**5**).

**Fig. 3:**
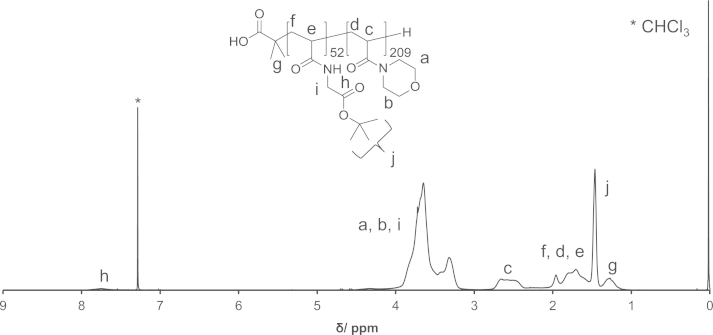
^1^H NMR spectrum of polymer (**6**).

**Fig. 4:**
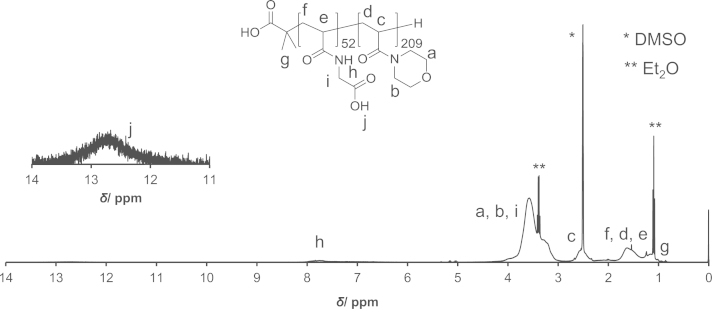
^1^H NMR spectrum of polymer (**7**).

**Fig. 5:**
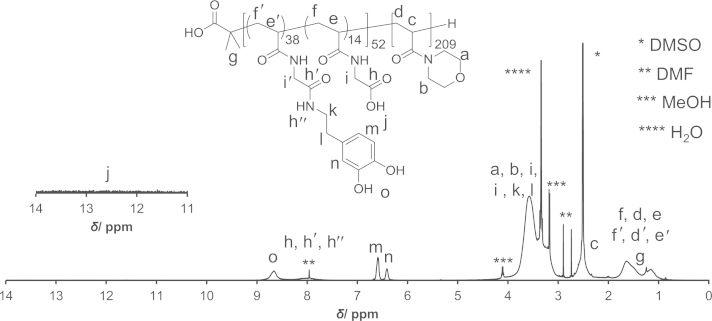
^1^H NMR spectrum of PAM-PDA polymer (**8**).

PAM-PDA block copolymers were synthesized as described in Ref [1]. Briefly, *N*-acryloyl glycine *tert*-butyl ester was polymerized by reversible addition-fragmentation chain transfer (RAFT) polymerization using 2-(dodecylthiocarbonothioylthio)-2-methylpropionic acid as the chain transfer agent (CTA) and AIBN as the initiator to yield poly(*N*-acryloyl glycine *tert*-butyl ester) (**3**). Thereafter, *N*-acryloyl morpholine was polymerized to yield the block copolymer (**5**). The CTA end group of polymer (**5**) was removed by radical-induced reduction to yield polymer (**6**). This polymer was treated with TFA/H_2_O to remove the *tert*-butyl ester groups (polymer (**7**)). Then, the polymer (**7**) was reacted with *N*-hydroxysuccinimide (NHS) in the presence of *N*,*N*′-dicyclohexylcarbodiimide (DCC) and thereafter reacted with dopamine (DA) to yield the PAM-PDA_*x*_ polymer (**8**). This reaction was carried out at different concentrations of DA, DCC and NHS as described in Table 1. The polymers were analyzed by ^1^H NMR (Figs. 1–5). The degree of DA modification was controlled by the feed ratio of carboxyl groups of polymer (**7**) and DA (Table 1).

### 1.2 Cyclic voltammograms of DA and the PAM-PDA micelles

**Fig. 6:**
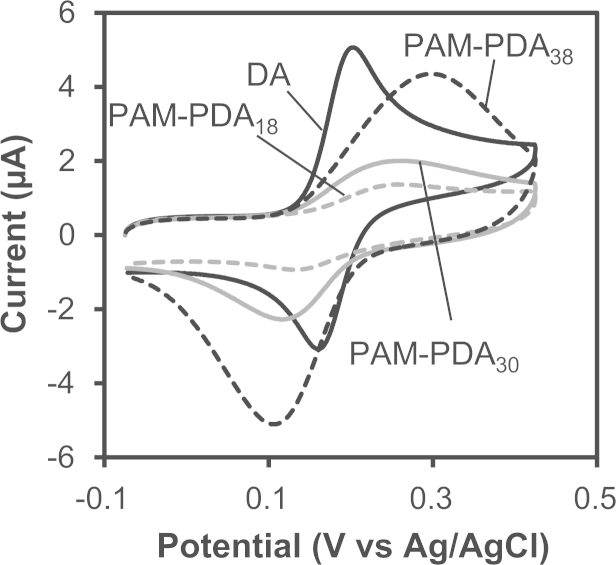
Cyclic voltammograms of DA and the PAM-PDA micelles in phosphate buffer (0.1 M, pH 7.0) at a scan rate of 100 mV/s.

**Fig. 7:**
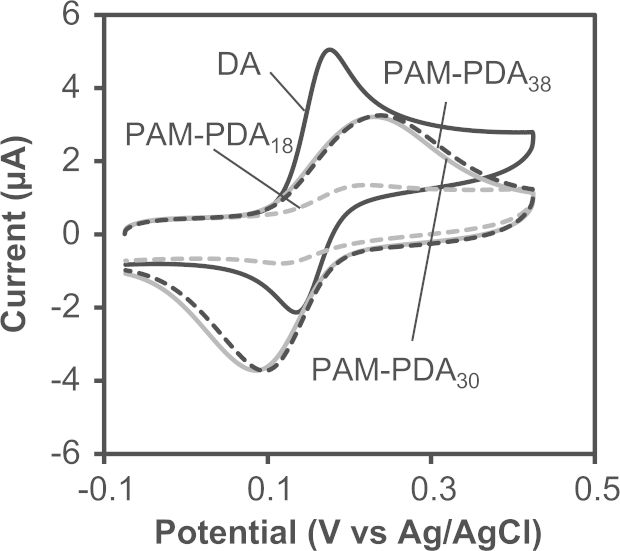
Cyclic voltammograms of DA and the PAM-PDA micelles in phosphate buffer (0.1 M, pH 7.4) at a scan rate of 100 mV/s.

DA and the PAM-PDA micelles were dissolved in 0.5 mL water at 2.0 mM of catechol moieties and mixed with 9.5 mL phosphate buffer (0.1 M, pH 7.0 and 7.4). The solutions were degassed by bubbling argon and kept at 25 °C using a thermostatic bath. Cyclic voltammograms were recorded on an ALS 600E electrochemical analyzer equipped with a CS-3A Cell Stand and a three-electrode set up: an Ag/AgCl/saturated KCl reference electrode, a glassy carbon (0.79 cm^2^) working electrode and a platinum wire counter electrode. The scan rate was 100 mV/s. Cyclic voltammograms at pH 7.0 and 7.4 are shown in Figs. 6 and 7, respectively. The peak potentials are summarized in Table 2 (pH 7.0) and Table 3 (pH 7.4).

### 1.3 Morphology of PAM-PDA_18_ micelles

**Fig. 8:**
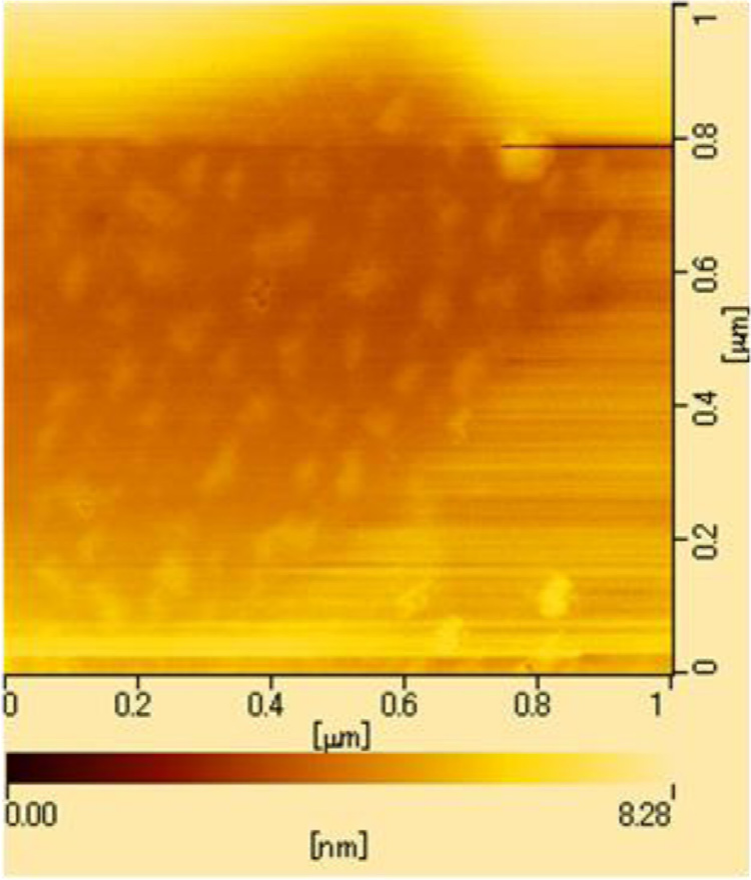
AFM image of the PAM-PDA_18_ micelles.

PAM-PDA_18_ micelles were adsorbed onto a fresh mica surface and air dried. The AFM image in Fig. 8 was obtained on a Seiko SPA400 in dynamic mode using a Si probe (SI-DF20, Seiko).

## Conflicts of interest

The authors declare no competing financial interests.

## Figures and Tables

**Table 1:** Synthetic details of PAM-PDA_*x*_ block copolymer synthesis.

	Carboxyl groups [M]	DA [M]	NHS [M]	DCC [M]	*x*
PAM-PDA_18_	0.07	0.07	0.07	0.07	18
PAM-PDA_30_	0.07	0.14	0.14	0.14	30
PAM-PDA_38_	0.07	0.21	0.21	0.21	38

**Table 2:** Electrochemical parameters of DA and the PAM-PDA micelles in phosphate buffer (0.1 M, pH 7.0).

	*E*_pa_^a^	*E*_pc_^b^	Δ*E*_*p*_^c^	*E*_1/2_^d^
DA	203	160	43	182
PAM-PDA_18_	257	135	122	196
PAM-PDA_30_	262	116	146	189
PAM-PDA_38_	298	106	192	202

a *E*_pa_: anodic peak potential.

b *E*_pc_: cathodic peak potential.

c Δ*E*_*p*_=*E*_pa_−*E*_pc_.

d *E*_1/2_=(*E*_pa_+*E*_pc_)/2.

**Table 3:** Electrochemical parameters of DA and the PAM-PDA micelles in phosphate buffer (0.1 M, pH 7.4).

	*E*_pa_^a^	*E*_pc_^b^	Δ*E*^c^	*E*_1/2_^d^
DA	176	135	41	156
PAM_209_-PDA_18_	222	117	105	170
PAM_209_-PDA_30_	231	86	145	159
PAM_209_-PDA_38_	239	95	144	167

a *E*_pa_: anodic peak potential.

b *E*_pc_: cathodic peak potential.

c Δ*E*_*p*_=*E*_pa_−*E*_pc_.

d *E*_1/2_=(*E*_pa_+*E*_pc_)/2.
